# *Prkci* Regulates Autophagy and Pancreatic Tumorigenesis in Mice

**DOI:** 10.3390/cancers14030796

**Published:** 2022-02-04

**Authors:** Kristin S. Inman, Yi Liu, Michele L. Scotti Buzhardt, Michael Leitges, Murli Krishna, Howard C. Crawford, Alan P. Fields, Nicole R. Murray

**Affiliations:** 1 Department of Cancer Biology, Mayo Clinic, Jacksonville, FL 32224, USA; kristin.inman@nih.gov (K.S.I.); liu.yi1@mayo.edu (Y.L.); michele.buzhardt@neogenomics.com (M.L.S.B.); hcrawfo1@hfhs.org (H.C.C.); fields.alan@mayo.edu (A.P.F.); 2 Environmental Health Perspectives/National Institute of Environmental Health Sciences, Durham, NC 27709, USA; 3 Neogenomics Laboratories, Clinical Division, Charlotte, NC 28104, USA; 4 Department of BioMedical Sciences, Faculty of Medicine, Memorial University, St. John’s, NL A1M 2V7, Canada; mleitges@mun.ca; 5 Department of Pathology/Lab Medicine, Mayo Clinic, Jacksonville, FL 32224, USA; Krishna.Murli@mayo.edu; 6 Department of Surgery, Henry Ford Pancreatic Cancer Center, Detroit, MI 48202, USA

**Keywords:** pancreatic cancer, tumor progression, protein kinase C iota, autophagy, immune cell infiltration

## Abstract

**Simple Summary:**

Pancreatic cancer is a highly fatal disease that does not respond well to current cancer therapies. We previously showed that protein kinase C iota (PKCι) is highly expressed in pancreatic cancer, and inhibition of PKCι signaling in human pancreatic cell lines blocks tumor growth. In this study, we used a genetic mouse model to investigate the role of PKCι in pancreatic cellular homeostasis and pancreatic tumorigenesis. We found that inhibition of PKCι expression in the mouse pancreas blocked autophagy, altered infiltration of immune cells, and prevented pancreatic cancer formation. Taken together, these findings reveal a promotive role for PKCι in pancreatic cancer development.

**Abstract:**

Protein kinase C iota (PKCι) functions as a bonafide human oncogene in lung and ovarian cancer and is required for *Kras^G12D^*-mediated lung cancer initiation and progression. PKCι expression is required for pancreatic cancer cell growth and maintenance of the transformed phenotype; however, nothing is known about the role of PKCι in pancreas development or pancreatic tumorigenesis. In this study, we investigated the effect of pancreas-specific ablation of PKCι expression on pancreatic cellular homeostasis, susceptibility to pancreatitis, and *Kras^G12D^*-mediated pancreatic cancer development. Knockout of pancreatic *Prkci* significantly increased pancreatic immune cell infiltration, acinar cell DNA damage, and apoptosis, but reduced sensitivity to caerulein-induced pancreatitis. *Prkci*-ablated pancreatic acinar cells exhibited P62 aggregation and a loss of autophagic vesicles. Loss of pancreatic *Prkci* promoted *Kras^G12D^*-mediated pancreatic intraepithelial neoplasia formation but blocked progression to adenocarcinoma, consistent with disruption of autophagy. Our results reveal a novel promotive role for PKCι in pancreatic epithelial cell autophagy and pancreatic cancer progression.

## 1. Introduction

Pancreatic ductal adenocarcinoma (PDAC) is one of the deadliest forms of cancer. With a 5-year survival rate of 10%, PDAC is predicted to result in over 48,000 deaths in the U.S. in 2021 [1]. This high mortality rate is attributable to late detection and poor response of PDAC to conventional therapies. Current treatment options elicit a limited therapeutic response, in most cases only prolonging patient survival by a matter of weeks to months [2]. The lack of durable therapeutic responses highlights the critical need to identify more effective therapeutic targets for the treatment of pancreatic cancer.

PDAC development is thought to progress from low grade dysplasia, pancreatic intraepithelial neoplasia 1-2 (PanIN1-2), to high grade dysplasia (PanIN3), before advancing to adenocarcinoma [3]. Greater than 90% of all PDACs contain an activating *KRAS* mutation, and expression of an oncogenic mutant of *Kras* (*Kras^G12D^*) in mouse pancreatic epithelium (*Lox-Stop-Lox-Kras^G12D^; Ptf1a^C^^re/+^*; *KC* genetically engineered mouse model; GEMM) recapitulates the full spectrum of human PDAC development [4,5,6,7,8,9]. *Kras*^G12D^-induced PanINs are detectable in the pancreas by 1-2 months of age as small focal lesions. PanIN lesion initiation and progression continue over the lifespan of the mouse with a small subset of the high grade PanINs progressing to pancreatic cancer [8]. The overall similarity of this model to human disease development makes the *KC* mouse a useful GEMM to assess the role of specific genes in pancreatic tumorigenesis and to evaluate potential therapeutic targets for the prevention and treatment of PDAC [9,10,11].

Atypical protein kinase C (PKC) iota, *PRKCI*, is highly expressed in numerous cancers [12] and is a bonafide human oncogene in lung and ovarian cancers [13,14]. Specifically, the protein product of *PRKCI*, PKCι, is overexpressed in a large percent of lung and ovarian cancers where high PKCι expression predicts poor survival, and disruption of PKCι signaling blocks cancer cell transformed growth and tumor formation [13,15,16,17,18]. We and others have shown that *PRKCI* is consistently elevated in PDAC compared to paired normal tissue [19,20], and that inhibition of PKCι expression in pancreatic cancer cell lines significantly reduces pancreatic cancer cell growth in vitro, and tumor growth, angiogenesis, and metastasis in vivo demonstrating that PKCι is required for the transformed phenotype of PDAC cells [20]. 

*Prkci* plays a promotive role in *Kras^G12D^*-mediated initiation of lung and colon carcinogenesis [12,16,21]. In the *Kras^G12D^*-driven, *KC* model of pancreatic tumorigenesis, PKCι expression is elevated in both early PanIN lesions and adenocarcinoma, similar to human disease [22]. In the current study, we used tissue-specific ablation of *Prkci,* the gene expressing murine PKCι, to investigate the role of PKCι in pancreas homeostasis, susceptibility to pancreatitis, and in the initiation and progression of pancreatic cancer. We report that mice lacking pancreatic PKCι expression developed normally but exhibited increased pancreatic apoptosis and immune cell infiltration and a lack of autophagy in pancreatic acinar cells. Consistent with the required role for autophagy in pancreatic cancer development, ablation of pancreatic PKCι blocked progression of early *Kras^G12D^*-mediated PanINs.

## 2. Materials and Methods

### 2.1. Mice

Floxed PKCiota (*Prkci^f/f^*) mice (previously described [23,24,25]) are maintained in the Mayo Clinic Florida barrier facility. *Ptf1a^Cre/+^* mice were a gift from Pinku Mukherjee, University of North Carolina [26]. *Prkci^f/f^* mice were crossed with *Ptf1a^Cre/+^* mice to generate *Ptf1a^Cre/+^; Prkci^f/+^* mice. These mice were then crossed to generate *Ptf1a^Cre/+^*, *Prkci^f/f^* (*Prkci*^∆*panc*^), and *Prkci^f/f^* mice.

*LSL-Kras^G12D^* mice were originally obtained from the NCI Mouse Repository. *LSL-Kras^G12D^* mice were crossed with *Ptf1a^Cre/+^* mice to generate *LSL-Kras^G12D^; Ptf1a^C^^re/+^* mice (*KC* mice), as described [8,27]. *LSL-Kras^G12D^; Prkci^f/f^* mice [13] were crossed with *KC* mice to generate *LSL-Kras^G12D^; Ptf1a^C^^re/+^*, *Prkci^f/f^* (*KCI*) mice. Recombination of the floxed alleles was confirmed by PCR analysis of genomic DNA as described [24]. Mice were housed in microisolator cages in a pathogen-free barrier facility and maintained at a constant temperature and humidity on a 12h light/12h dark cycle. Mice were provided a standard irradiated rodent chow (PicoLab Mouse Diet 20 5058; LabDiet, St. Louis, MO, USA) and filtered water ad libitum throughout the experimental protocol unless otherwise specified. All mice were visually checked daily for body condition, and mice on the experimental protocol were weighed twice weekly. All mice were euthanized by CO_2_ inhalation according to the American Veterinary Medical Association Guidelines. All animal experiments and procedures performed were approved by the Mayo Clinic Institutional Animal Care and Use Committee (IACUC protocols A48510; A72513, A00002363).

To analyze tissue proliferation, one hour prior to harvest, mice were injected intraperitoneally with 50 ug/kg 5-bromo-2′-deoxyuridine (BrdUrd; Sigma-Aldrich, St. Louis, MO). At harvest, mice were weighed and the pancreata were removed, blotted dry on filter paper and weighed, and fixed in 10% formalin for H&E and immunohistochemical (IHC) analysis. 

Differences in survival between genotypes, and between sexes within a genotype, were analyzed by Kaplan-Meier survival analyses performed using Graphpad Prism. Survival was defined as the age when a mouse died, was moribund, and/or met IACUC-defined humane endpoints for euthanasia, such as weight loss, decreased activity, body condition score, or behavioral changes.

### 2.2. Mouse Models of Caerulein-Induced Pancreatitis

To induce acute pancreatitis, 1–2 month old mice were injected intraperitoneally with 50 µg/kg caerulein (American Peptide, Sunnyvale, CA, USA) hourly for 8 hr and harvested 1 hr after the last caerulein injection [28]. To induce severe acute pancreatitis, 1–2 month old mice were injected intraperitoneally with 250 µg/kg caerulein twice daily for two weeks and harvested 24 h after the last caerulein injection [29]. Note that although this dosing regimen has been previously referred to as “chronic pancreatitis” [29], in true clinical chronic pancreatitis, the tissue does not heal, with regions of acinar tissue replaced with persistent acinar-to-ductal metaplasia (ADM), fibrosis, and inflammation. In this two-week cerulein-induced model of pancreatitis, the tissue is restored to histologically normal tissue within a week after ceasing the caerulein treatments. We therefore now refer to this treatment as “severe acute pancreatitis”. Mice were weighed immediately before euthanasia and blood was drawn immediately after euthanasia. For serum collection, blood was drawn from the posterior vena cava immediately after euthanasia, coagulated at room temperature for 30 min and serum was collected by centrifuging for 10 min at 10,000× *g*. Serum amylase was measured using the Phadebas amylase test (Magle Life Sciences, Cambridge, MA, USA) according to the manufacturer’s instructions. After serum removal, the pancreata was removed, blotted on a paper towel, weighed, and immediately fixed in 10% formalin for H&E, Masson’s trichrome staining, and IHC analysis. 

### 2.3. Immunoblot, Histological, and IHC Analyses

PKCι expression was detected by immunoblot analysis of protein extracted from the whole pancreas of 1.5 month old mice [30]. Pancreas tissues were processed for histology, aligned, and embedded for sectioning, and approximately 100 µm of tissue was removed to reach a full cross-section of the pancreas. Sections measuring 5 µm were then cut and stained by IHC as described previously [31]. One slide of pancreas tissue/mouse (typically 5–6 mice/group; see Figure legends for group sizes) was subjected to IHC detection of each antigen of interest. Group size was dependent on the number of available pancreas tissues at the time the analysis was performed. Stained slides were scanned at 20× magnification using Aperio ScanScope (Leica Biosystems, Buffalo Grove, IL, USA) to produce digital images. Aperio Imagescope software was used to annotate the digital image of the pancreas for quantitative analysis, excluding artifacts and non-pancreatic tissue. In some cases, an individual slide was stained either too dark or too light for automated analysis to be performed accurately, and therefore, that slide was not included in the analysis. The annotated area of the pancreas, typically a minimum area of 7–10 mm^2^/slide, or in the case of nuclear staining, a minimum of ~30,000 cells/slide, was analyzed. For the quantitation of antigen detection in PanIN lesions, only the PanIN-containing area of tissue was annotated for analysis. Quantitative analysis was performed using the Spectrum software IHC Nuclear V1 algorithm or IHC Positive Pixel count V9 algorithm, and data are presented as the percent positive stained nuclei, or percent positive staining area, respectively. All available data are presented, and no data points were eliminated as outliers.

H&E stained slides were used to evaluate edema and acinar cell damage (calculated as % non-normal or ADM histology) for pancreatitis damage score calculation [32]. H&E stained pancreas from 18 month old *KC* and *KCI* was evaluated by a clinical pathologist (M.K.) to determine the highest grade of tumor present using well-described criteria [8]. Antibody information is provided in Table S1. 

### 2.4. Reactive Oxygen Species Assay

Pancreata were harvested from 1.5 month old mice and quick-frozen in an Optimal Cutting Temperature compound (Sakura). Sections measuring 6 µm were incubated with 30 uM dihydroethidium (DHE; Molecular probes) for 8 min. Sections were then mounted with Vectrashield hard mounting media containing DAPI and imaged immediately by fluorescence microscopy.

### 2.5. Transmission Electron Microscopy

Six week old mice were fasted overnight, anesthetized with 100 mg/kg ketamine/10 mg/kg xylazine, and perfused with fixative (2% paraformaldehyde with 2.5% glutaraldehyde in phosphate-buffered saline, pH 7.2) via the vena cava. Death was confirmed by opening the chest cavity. The pancreata were removed and immersed in fixative at 4˚C until processed (at least 12 h). The tissue was rinsed with 0.1 M phosphate buffer and secondarily fixed in 1% osmium tetroxide followed by 2% aqueous uranyl acetate. Tissue was dehydrated in sequential ethanol washes followed by 100% acetone and embedded in Embed 812/Araldite epoxy resin and polymerized for 24 h at 60 °C. Ultrathin sections (0.1 micron) were post-stained with 2% lead citrate. Micrographs were obtained using a JEOL 1400 Plus transmission electron microscope (Tokyo, Japan) at 80 kV equipped with a Gatan Orius 4K camera (Pleasanton, CA, USA).

### 2.6. Rapamycin Treatment

Five to six week old mice were injected intraperitoneally with 0.2 mg/kg rapamycin or diluent (0.08% ethanol) 6 days/week for 2 weeks. Mice were harvested and the pancreas fixed in 10% buffered formalin and processed for IHC analysis.

### 2.7. Analysis of TCGA-PDAC Gene Expression and Patient Survival Data

Computational Analysis: RNA-seq data, gene-level copy number alterations estimated by the Genomic Identification of Significant Targets In Cancer (GISTIC), and survival data for the primary pancreatic ductal adenocarcinoma tumors in the Pancreatic Adenocarcinoma (PanCancer Atlas; PDAC) dataset were downloaded from the Cancer Genome Atlas (TCGA) data portal (http://www.cbioportal.org/public-portal/; RNA-seq data last accessed 30 January 2020 and survival data last accessed 11 November 2019) [33,34].

Gene Signature Score Analysis of PDAC: The gene signature score represents the principal component analysis (PCA) of the expression matrix of the PKCι-ECT2 signature. The gene/pathway signature score was applied using the built-in R prcomp and princomp functions. The PKCι-ECT2 pathway signature consists of the 10 ribosomal RNA processing genes that most highly correlate with *PRKCI* and *ECT2* expression in the TCGA PDAC dataset. The Spearman correlation coefficient and *p*-value are listed in Figure S9B. The autophagy risk score consists of the 10 autophagy-related genes with a prognostic value in PDAC and was constructed as a risk model based on the expression of these genes [35]. The risk score was calculated using the following formula: risk score = (0.1063 × CASP4) + (0.0416 × CHMP2B) + (0.0297 × EIF4G1) − (0.0498 × GABARAP) + (0.0983 × NCKAP1) − (0.2125 × PELP1) − (0.1453 × RAB24) − (0.3239 × RPTOR) + (0.0078 × TNFSF10) − (0.1622 × WIPI2).

Ingenuity Pathway Analysis (IPA): To identify genomic signatures associated with *PRKCI* expression in human PDAC tumors, we performed IPA on the TCGA PDAC dataset consisting of 146 primary PDAC tumors. Genomic data from tumors corresponding to the 25% of PDAC tumors with the highest *PRKCI* mRNA levels (Top25; *n* = 36) and those corresponding to the 25% of tumors with the lowest *PRKCI* expression (Btm25; *n* = 36) were compared. Genes that were differentially expressed with significance (*p*-value < 0.05; fold change > 1.5) between these groups were subjected to IPA. The core IPA analysis was performed using default settings: direct and indirect relationships between molecules supported by experimentally observed data were considered, networks did not exceed 35 molecules, and all sources of data from human studies in the Ingenuity Knowledge Base were considered. This generated priority lists for canonical pathways. Score values were calculated from hypergeometric distribution and right-tailed Fisher’s exact test. Canonical pathways were further filtered by −log(*p*-value) > 1.3 and Z-score > 1.3 or <−1.3.

Survival Analysis: PDAC patients surviving >30 days were stratified by *PRKCI* expression and segregated into high *PRKCI* and low *PRKCI* expression groups optimized for survival outcome based on an optimized cutoff value of *PRKCI* expression calculated using the R survival package [36]. The optimal cutoff was defined as the point with the most significant statistical difference in overall survival (log-rank test) using the R functions survfit and coxph. To assess whether the presence of *PRKCI* copy number gains (CNGs) was prognostic for PDAC patient survival, PDAC patients with *PRKCI* CNGs, and without *PRKCI* CNGs, were compared for overall survival. 

### 2.8. Statistical Analysis

Individual unpaired *t*-tests, without correction for multiple comparisons, with alpha = 5% were used to compare positive immunostaining between *Prkci^f/f^* and *Prkci*^∆panc^ groups and *KC* and *KCI* groups, and to compare the zymogen granules per cell between groups. Two-way analysis of variance with Bonferroni’s multiple comparisons test was used to compare pancreas/body weight and serum amylase of WT and *Prkci*^∆panc^ mice treated with saline or caerulein, and the percent of abnormal pancreas in *KC* and *KCI* mice. Fisher’s exact test was used to compare the presence of damage following induction of severe acute pancreatitis in WT and *Prkci* ^∆panc^ mice and PanIN progression in *KC* versus *KCI* mice. A chi-square test was used to assess the significance of the distribution of PDAC-TCGA tumors with and without CNGs into high *PRKCI* and low *PRKCI* groups. The log-rank (Mantel-Cox) test was used to compare survival curves. Unless otherwise stated, statistical analyses were performed using Graphpad Prism. Unless otherwise stated, *p* ≤ 0.05 was considered statistically significant. 

## 3. Results

### 3.1. Pancreas-Specific Ablation of Prkci Increased Apoptosis, Cellular Stress, and Immune Cell Infiltration

**Figure 1:**
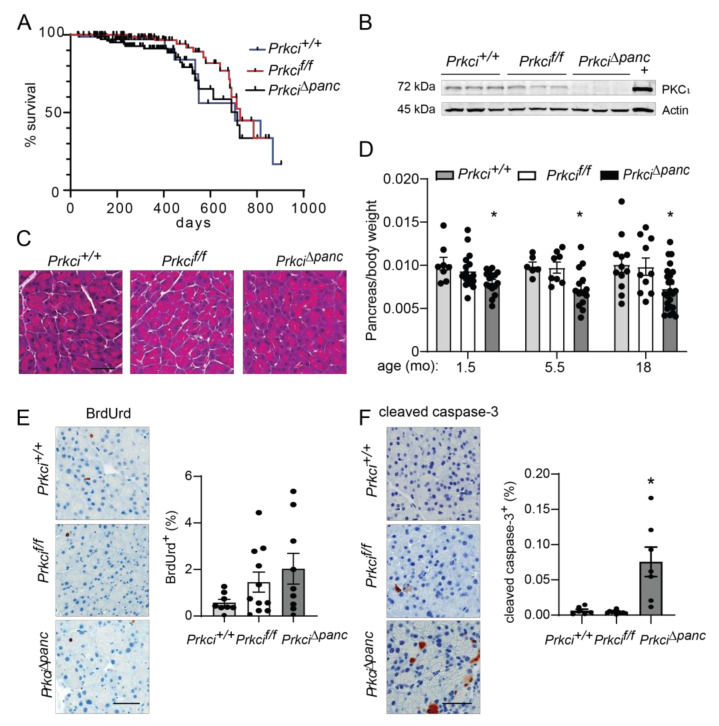
Characterization of pancreas-specific *Prkci* knockout mice. (**A**) Kaplan-Meier analysis of the overall survival of the control (*Prkci*^+/+^), *Prkc^f/f^,* and *Prkci*^∆*panc*^ mice is plotted. *n* = 81, 97, and 112, respectively. (**B**) Immunoblot analysis of PKCι protein expression in mouse pancreas (3 representative mice/genotype). Full immunoblots are in Figure S2. (**C**) Representative H&E stained pancreas from each genotype (3.5 month old). Scale bar = 50 µm. (**D**) The ratio of pancreas weight/body weight at harvest at indicated ages is plotted. Representative images of IHC detection and quantitative analysis of IHC staining of (**E**) cellular proliferation (BrdUrd incorporation) and (**F**) apoptosis (cleaved caspase-3) in mouse pancreas. Analyses were performed on mice harvested at 1.5 months of age, except as otherwise described. Scale bars = 50 µm. Quantification of pancreas weight/body weight ratio, cleaved caspase-3 expression, and BrdUrd incorporation is plotted as mean ± SE; *n* ≥ 6 mice/group. * *p* < 0.05 compared to *Prkci*^+/+^ and *Prkci^f/f^*.

**Figure 2:**
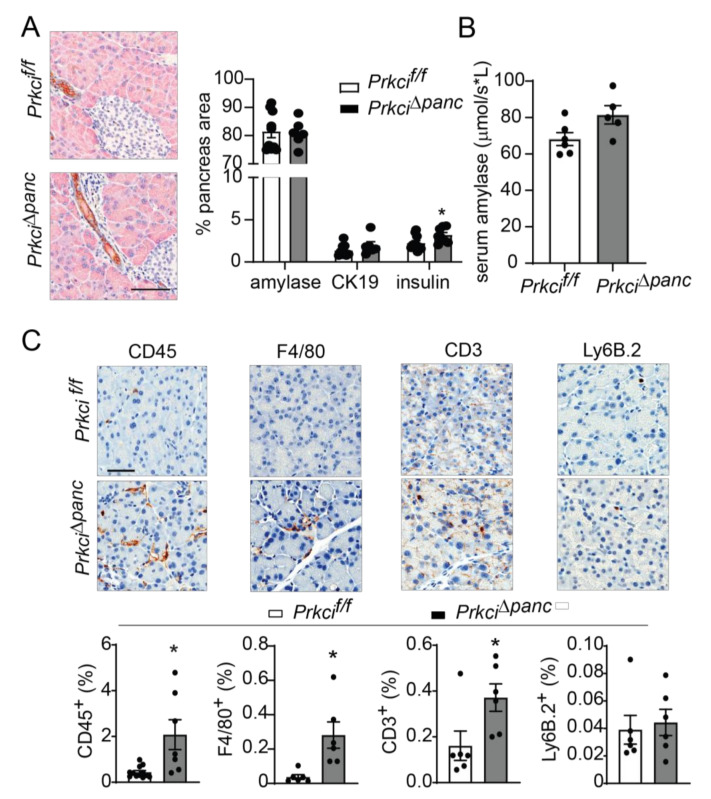
Ablation of pancreatic *Prkci* expression increases pancreas immune cell infiltration. (**A**) Representative image of IHC detection of acinar (amylase; pink) and ductal (cytokeratin-19, CK-19; brown) cell markers in the pancreata of 1.5 month old mice (scale bar = 100 µm; left panel). Quantitation of area of IHC detection of amylase, CK19, and insulin is plotted as mean ± SE; *n* ≥ 6 (right panel). (**B**) Basal serum amylase level in 3.5 month old mice (*n* = 6, 5, respectively) is plotted as mean ± SE. (**C**) Representative images of IHC detection of infiltrating leukocytes (CD45^+^), macrophages (F4/80^+^), T-cells (CD3^+^), and neutrophils (Ly6.B^+^) in pancreata of 1.5 month old mice (scale bar = 50 µm). Quantitative analysis of IHC staining is plotted below images as mean ± SE; *n* ≥ 6. * *p* < 0.05 compared to *Prkci^f/f^*.

Floxed *Prkci* (*Prkci^f/f^*) mice [25] were crossed with mice expressing Cre recombinase driven by the *Ptf1a* promoter (*Ptf1a^Cre/+^* mice) [26] to generate mice with pancreas-specific ablation of *Prkci* (*Prkci*^∆*panc*^ mice). *Prkci*^∆*panc*^ mice, and mice with unrecombined floxed *Prkci* alleles (*Prkci^f/f^* mice), were born at the predicted Mendelian frequency and exhibited no significant difference in overall survival compared to control mice (*Prkci*^+/+^, Figure 1A; see also Figure S1). Immunoblot analysis confirmed loss of PKCι protein expression in the *Prkci*^∆*panc*^ pancreas (Figure 1B, see also Figure S2). While ablation of pancreatic *Prkci* did not alter the overall morphology of the pancreas tissue (Figure 1C), *Prkci*^∆*panc*^ mice exhibited a significantly lower pancreas weight, as determined by a smaller ratio of pancreas weight to body weight, that persisted over time (Figure 1D). This result was not attributable to a significant alteration in body weight (Figure S3) or acinar cell proliferation (Figure 1E). Instead, the difference in pancreas weight was likely driven by the significantly higher level of apoptosis detected in *Prkci*^∆*panc*^ pancreatic acinar cells, compared to *Prkci*^+/+^ and *Prkci^f/f^* pancreatic acinar cells (Figure 1F). Consistent with the pancreatic histology shown in Figure 1C, the distribution of amylase expressing- (acinar), cytokeratin 19-expressing (ductal), and insulin-expressing (endocrine) cells was similar in *Prkci^f/f^* and *Prkci*^∆*panc*^ pancreas, although insulin-staining cells were detected at a slightly, but significantly higher level in *Prkci*^∆*panc*^ pancreas (Figure 2A). Importantly, despite a higher level of acinar cell apoptosis, *Prkci*^∆*panc*^ mice did not exhibit a significantly lower percent of amylase-expressing acinar cells (Figure 2A) or a significantly higher level of serum amylase, an indicator of acinar cell damage (Figure 2B).

### 3.2. Prkci Loss Protected against Caerulein-Induced Pancreatic Damage

We next assessed whether the elevated level of pancreatic acinar cell apoptosis in *Prkci*^∆*panc*^ mice was associated with altered pancreatic immune cell infiltration. Immunohistochemical (IHC) detection of CD45^+^ cells revealed a significantly higher level of immune cells in *Prkci*^∆*panc*^ pancreas compared to *Prkci^f/f^* pancreas (Figure 2C). Characterization of the immune cell subtypes present in the *Prkci*^∆*panc*^ mouse pancreas revealed a significantly higher level of macrophages (F4/80^+^) and T cells (CD3^+^), but no difference in neutrophil infiltration (Ly6B.2^+^; Figure 2C).

**Figure 3:**
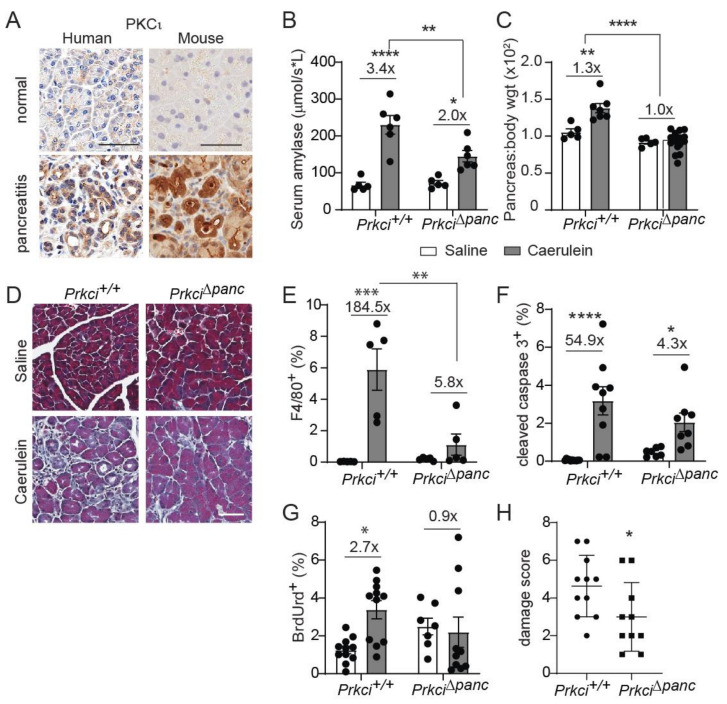
Ablation of pancreatic *Prkci* expression protects against caerulein-mediated damage. (**A**) Representative images of IHC detection of PKCι expression in normal human and mouse pancreas, human pancreatitis, and caerulein-induced severe acute mouse pancreatitis (scale bar = 50 µm). See additional images of pancreatitis tissues in Figure S4. Patient pancreatitis tissue, representative of *n* = 4. (**B**) Serum amylase in mice with caerulein-induced acute pancreatitis. (**C**) Pancreas/body weight ratio in 1.5 month old mice with caerulein-induced acute pancreatitis. (**D**) Representative images of Masson’s trichrome stain in mouse pancreas after caerulein-induced severe acute pancreatitis (scale bar = 50 µm). Quantitation of IHC detection of (**E**) macrophages (F4/80^+^), (**F**) apoptosis (cleaved caspase 3), and (**G**) proliferation (BrdUrd incorporation) in pancreata of mice with caerulein-induced severe acute pancreatitis. (**H**) Pancreatitis damage score plotted for mice with caerulein-induced severe acute pancreatitis. (**B**,**C**,**E**–**H**) Mean ± SE is plotted; *n* ≥ 5 for acute and *n* ≥ 7 for severe acute pancreatitis analyses; * *p* < 0.05, ** *p* < 0.01, *** *p* < 0.001, **** *p* < 0.0001. The number above bar indicates fold-change induced by caerulein treatment.

Elevated pancreatic immune cell infiltration is also observed in severe acute pancreatitis [37,38]. PKCι expression was higher in clinical pancreatitis than in normal pancreas (Figure 3A), suggesting a potential role for PKCι in the response of acinar cells to pancreatitis-inducing stimuli. Therefore, we examined the effect of loss of pancreatic PKCι expression on susceptibility to pancreatitis. Caerulein, a cholecystokinin ortholog, promotes intracellular activation of digestive enzymes normally secreted by acinar cells, resulting in acinar cell damage and an inflammatory response that resembles acute or severe acute pancreatitis, depending on the dose and timing of caerulein administration [28,39]. Consistent with the human disease, PKCι expression was also higher in pancreata exhibiting caerulein-induced severe acute pancreatitis than in pancreata of control-treated mice (Figure 3A, see also Figure S4). One day of repeated caerulein injections induced acinar cell damage and pancreatic edema in *Prkci*^+/+^ mice, detected as a significantly higher level of serum amylase (Figure 3B) and pancreas weight (Figure 3C), respectively, similar to the pathology of acute pancreatitis in humans [28,40]. In the absence of caerulein-treatment, loss of PKCι expression did not result in histological alterations consistent with pancreatitis (Figure 3D), even in 18 month old mice (Figure S5). *Prkci*^∆*panc*^ mice were partially, but significantly, protected from an acute pancreatitis-induced increase in serum amylase (Figure 3B), and this treatment did not induce an increase in pancreas/body weight ratio in *Prkci*^∆*panc*^ mice (Figure 3C), suggesting that loss of pancreatic PKCι expression reduced susceptibility to caerulein-induced acinar cell damage.

We next investigated the effect of ablation of pancreatic *Prkci* on the development of severe acute pancreatitis. *Prkci*^+/+^ mice injected with caerulein twice daily for two weeks exhibited signs of severe acute pancreatitis, including fibrosis, edema, dilated acinar lumina, and ADM [29], while *Prkci*^∆*panc*^ mice exhibited less pancreatic damage (Figure 3D). Furthermore, this two-week caerulein treatment induced a significant influx of macrophages into *Prkci*^+/+^ mouse pancreas (Figure 3E), which is characteristic of severe acute pancreatitis [37]. In contrast, the same two-week caerulein treatment did not induce a significant increase in the level of macrophages in *Prkci*^∆*panc*^ mouse pancreata, compared to control-treated *Prkci*^∆*panc*^ mouse pancreata (Figure 3E). Severe acute pancreatitis significantly increased both apoptosis and proliferation in *Prkci*^+/+^ mouse pancreata (Figure 3F,G). In contrast, severe acute pancreatitis induced a significant increase in apoptosis in the pancreata of caerulein-treated *Prkci*^∆*panc*^ mice, but proliferation was not significantly increased, suggesting less overall injury (Figure 3F,G). Finally, calculation of an overall pancreatitis damage score, using a previously described formula incorporating edema, immune infiltration, and acinar cell damage [32], confirmed significantly less pancreatic damage in *Prkci*^∆*panc*^ mice compared to *Prkci*^+/+^ mice (Figure 3H). These results reveal that, despite a baseline of higher immune cell infiltration and apoptosis, *Prkci*^∆*panc*^ mice were protected against caerulein-induced pancreatitis.

### 3.3. Loss of Pancreatic Prkci Expression Promoted Pancreatic Oxidative Stress and DNA Damage and Blocked Acinar Cell Autophagy

**Figure 4:**
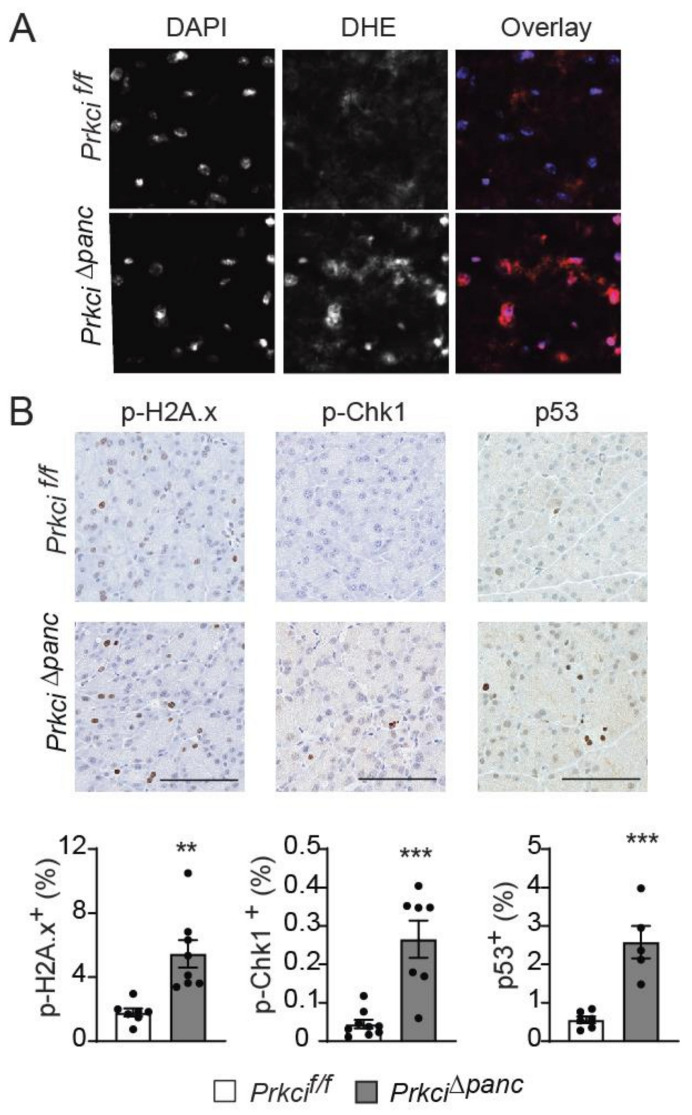
Loss of pancreatic PKCι expression induces oxidative stress and DNA damage. (**A**) Representative images of superoxide detected by DHE staining and overlaid with DAPI staining in pancreata of 1.5 month old *Prkci^f/f^* and *Prkci*^∆*panc*^ mice. (**B**) IHC detection of p-H2A.x, p-Chk1, and nuclear p53 in 1.5 month old *Prkci^f/f^* and *Prkci*^∆*panc*^ mice pancreata. Bars = 100 µm. Quantitative analysis of IHC staining is presented in the graphs below images. Mean ± SE is plotted; *n* ≥ 6. ** *p* < 0.01, *** *p* < 0.001.

To better understand the cellular effects of pancreatic *Prkci* ablation*,* we assessed the level of basal cellular stress in the pancreas of *Prkci*^∆*panc*^ and *Prkci^f/f^* mice. Reactive oxygen species were higher in the pancreas of *Prkci*^∆*panc*^ mice compared to *Prkci^f/f^* mice (Figure 4A). *Prkci*^∆*panc*^ mice also had significantly higher levels of phosphorylated H2A.x (p-H2A.x; a sensitive marker of DNA damage) [41], p-Chk1 (an indicator of activation of DNA damage response), and nuclear p53 (an indicator of the cellular stress response) staining in acinar cells (Figure 4B), suggesting that loss of pancreatic *Prkci* expression leads to acinar cell DNA damage and subsequent p53 activation, likely driving the elevated apoptosis observed in *Prkci*^∆*panc*^ acinar cells (Figure 1E). 

**Figure 5:**
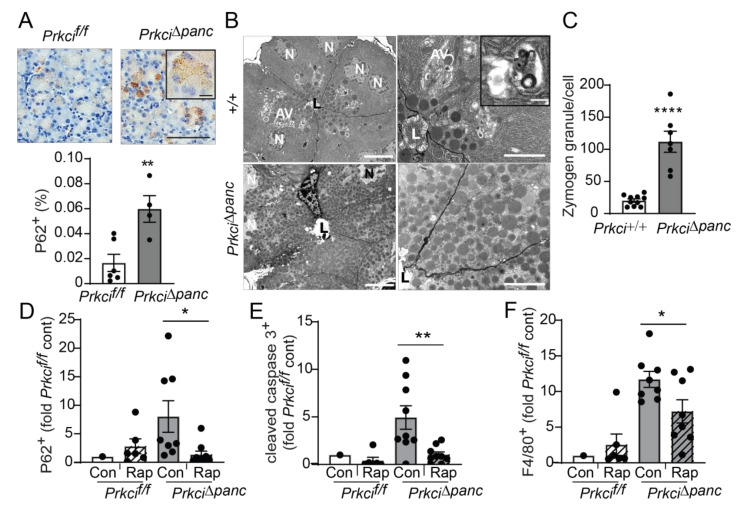
Pancreas-specific *Prkci* ablation blocks acinar cell autophagy. (**A**) IHC detection of P62 aggregates in pancreata of representative 1.5 month old *Prkci^f/f^* and *Prkci*^∆*panc*^ mice (scale bar = 100 µm, scale bar inset = 15 µm). Quantitative analysis of IHC staining is plotted below images as mean ± SE; *n* = 4–7. (**B**) Representative images of transmission electron microcopy of pancreatic epithelial cells of 6 week old *Prkci^+/+^* and *Prkci*^∆*panc*^ mice at low (left images; scale bar = 10 µm) and high magnification (right images; scale bar = 2 µm). Inset shows a single membrane autophagic vesicle (scale bar = 333 nm). Lumen (L), nucleus (N), and autophagic vesicles (AV) are identified. (**C**) Quantitation of zymogen granules/cell is plotted as mean ±  SE; *n* = 7. Control- and rapamycin-treated *Prkc^f/f^* and *Prkci*^∆*panc*^ mice were analyzed by IHC for (**D**) P62 aggregates, (**E**) cleaved caspase-3, and (**F**) F4/80 expression in mouse pancreas. Quantitative analysis of IHC staining is plotted as mean ±  SE. *n* = 7–9/group; * *p* < 0.05, ** *p* < 0.01, **** *p* < 0.0001.

Macroautophagy (autophagy) is a lysosome-mediated cellular process that degrades long-lived proteins and organelles. Autophagy is activated by DNA damage and is required for cellular responses to DNA damage (reviewed in [42]). Mice with a genetic blockade of acinar cell autophagy exhibit higher basal levels of indicators of cellular stress, including apoptosis, immune cell infiltration, and nuclear p53 expression [9,43], similar to the phenotype we observed in *Prkci*^∆*panc*^ mice (Figure 1,Figure 2,Figure 4). In addition, we observed significantly more cytoplasmic aggregates of P62 in *Prkci*^∆*panc*^ pancreata compared to *Prkci^f/f^* pancreata (Figure 5A). Electron microscopic analysis of the pancreata of fasted mice revealed that autophagic vesicles were readily discernible in *Prkci*^+/+^ mouse pancreata but not in *Prkci*^∆*panc*^ pancreata, and *Prkci*^∆*panc*^ acinar cells contained significantly more zymogen granules than control acinar cells (Figure 5B,C). Since both P62 and intracellular zymogen granules are degraded by autophagy [44], these observations are consistent with a blockade of autophagy in *Prkci*^∆*panc*^ acinar cells.

To confirm that the higher level of P62 aggregates detected in *Prkci*^∆*panc*^ pancreas was due to blocked autophagy, we used the mTORC1 inhibitor, rapamycin, to de-repress mTORC1-mediated inhibition of autophagy [45]. As expected, activation of autophagy by mTORC1 inhibition resulted in significantly less accumulated P62 aggregates in *Prkci*^∆*panc*^ acinar cells, without a significant effect on the already low levels of P62 aggregates in *Prkci^f/f^* acinar cells (Figure 5D), supporting the conclusion that loss of *Prkci* expression blocks acinar cell autophagy. Additionally, rapamycin treatment of *Prkci*^∆*panc*^ mice resulted in a significantly lower level of acinar cell apoptosis and pancreatic macrophage infiltration (Figure 5E,F), consistent with the well-characterized role of autophagy in protecting against inflammation and tissue damage (reviewed in [46]). 

### 3.4. Ablation of Pancreatic Prkci Promoted PanIN Formation but Blocked Progression to PDAC

**Figure 6:**
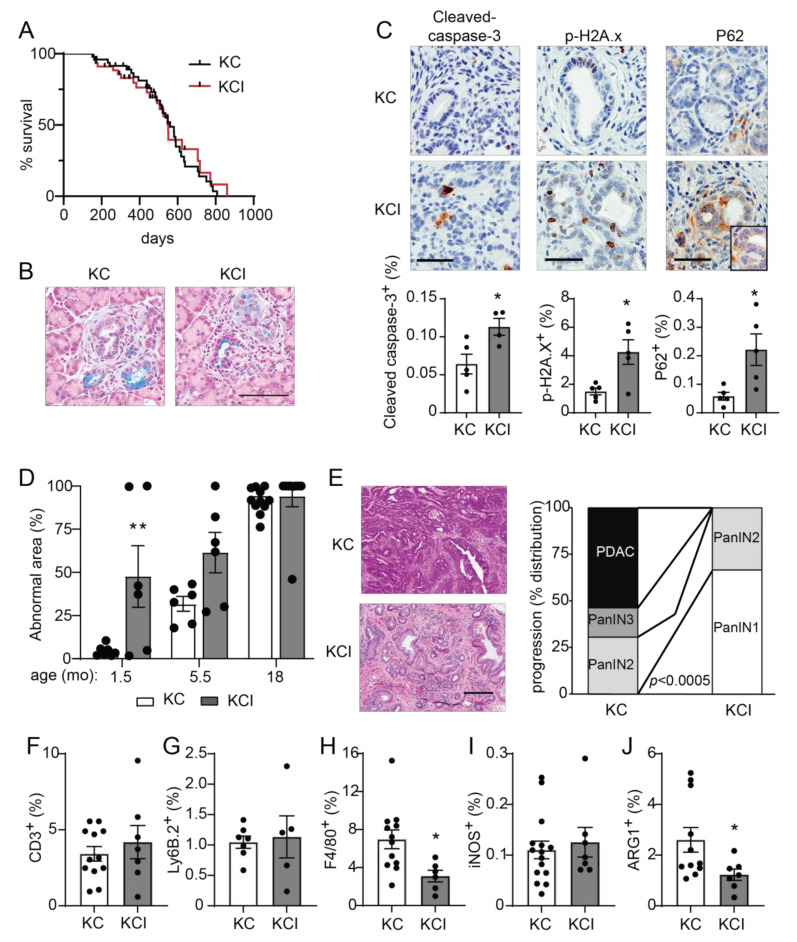
Pancreas-specific *Prkci* knockout increases early *Kras^G12D^*-mediated PanIN formation, but blocks progression to adenocarcinoma and alters immune cell infiltration. (**A**) Kaplan-Meier analysis of overall survival of *KC* and *KCI* mice (*n* = 51, 50, respectively). (**B**) Representative images of Alcian blue-stained pancreas from 1.5 month old *KC* and *KCI* mice (scale bar = 100 µm). (**C**) Representative images (top) and quantitative analysis of IHC detection (bottom) of cleaved caspase-3, p-H2A.x, and P62 aggregates in PanIN lesions of 1.5 month old *KC* and *KCI* mice, (scale bar = 50 µm). Results plotted as mean ± SE; *n* = 4–5; * *p* < 0.05. (**D**) Quantitation of area of abnormal tissue (ADM, dysplasia, and PDAC) in *KC* and *KCI* mouse pancreas is plotted; *n* ≥ 6, ** *p* < 0.01 compared to *KC*. (**E**) Representative images of H&E stained pancreas from 18 month old *KC* and *KCI* mice are shown (left; scale bar = 200 µM). Neoplastic progression of 18 month old *KC* and *KCI* mice is plotted; (right; *n* = 13, 9; see also Figure S7D). Quantitative analysis of IHC detection of (**F**) T cells (CD3^+^), (**G**) neutrophils (Ly6.B^+^), (**H**) macrophages (F4/80^+^), (**I**) M1 polarized macrophages (iNOS^+^), and (**J**) M2 polarized macrophages (ARG1^+^) in pancreatic lesions of 18 month old *KC* and *KCI* mice. IHC quantitation is plotted as mean ± SE; *n* = 6–15. * *p* < 0.05.

We have previously described a required role for PKCι in the transformed growth of pancreatic cancer cells via activation of an oncogenic RAC-MEK-ERK signaling pathway, and we showed that high PKCι protein expression correlated with poor survival in PDAC patients [20]. PKCι expression is elevated in *Kras^G12D^*-induced early metaplastic lesions in the mouse pancreas and in human PanINs [22,47], and oncogenic *KRAS*-induced PKCι expression in transformed pancreatic epithelial cells [48], suggesting that PKCι may play a promotive role in the early stages of pancreatic carcinogenesis. To address this question, we generated mice with *Kras^G12D^*-driven pancreatic cancer (*KC* GEMM) [8,27] and knockout of pancreatic *Prkci* by crossing *Prkci^f/f^* mice with *KC* mice to generate *LSL-Kras^G12D^; Ptf1a^C^^re/+^*; *Prkci^f/f^* (*KCI*) mice. *KCI* mice were born at the predicted Mendelian frequency and exhibited no significant difference in survival compared to *KC* mice (median survival 550 days vs 560 days, respectively; Figure 6A; see also Figure S6A,B). At 1.5 months of age, areas of pancreatic ADM and early PanIN lesions could be detected in both *KC* and *KCI* mice (Figure 6B). PKCι drives a RAC-MEK-ERK signaling pathway in human PDAC cells, and both PKCι and p-ERK are expressed at a high level in human PDAC [20]. To assess whether PKCι also activates MEK-ERK signaling in the *KC* mouse model, we performed IHC for p-ERK on *KC* and *KCI* PanIN lesions. *KC* PanIN lesions expressed high levels of PKCι and p-ERK compared to *KCI* lesions (Figure S6C), suggesting that PKCι regulates ERK activation in mouse PanINs. *KCI* PanIN lesions exhibited significantly more apoptosis, DNA damage, and P62 aggregates compared to PanINs in *KC* mice (Figure 6C). Electron microscopy showed a lack of autophagic vesicles (Figure S7A) and significantly more zymogen granules in *KCI* versus *KC* pancreatic cells (*p* < 0.05; Figure S7B), indicating a block in autophagy in *KCI* mouse pancreas. 

We next assessed the effect of pancreatic *Prkci* ablation on the timing of *Kras^G12D^*-induced PanIN development and progression to pancreatic cancer. As previously described [8], the pancreata of *KC* mice are significantly larger than those of control mice (as measured by pancreas/body weight ratio), and this difference became more pronounced as the mice aged (Figure S7C). In contrast, the pancreas/body weight ratio for *KCI* mice remained comparable to similarly aged, control (*Prkci^f/f^)* mice, up to 18 months of age (Figure S7C). As described [8], the pancreata of young *KC* mice were comprised of mostly normal pancreatic tissue (Figure 6D, 1.5 month old). Normal acinar cells were gradually replaced by dysplasia until only ~5% of the normal pancreatic tissue remained by 18 months of age (Figure 6D). The time course of the conversion of a normal pancreas to dysplasia was significantly different for *KCI* mice, with almost 50% of the *KCI* mouse normal pancreas already replaced by dysplasia by 1.5 months of age, and the remaining normal pancreas tissue progressively lost as the mice aged (Figure 6D). To assess pancreatic lesion progression, H&E stained pancreata from 18 month old *KC* and *KCI* mice (Figure 6E) were evaluated for the presence of low grade dysplasia (PanIN1-2), higher grade dysplasia (PanIN3), and adenocarcinoma (see Figure S7D). Over half (54%) of the *KC* mice had developed adenocarcinoma by 18 months of age (Figure 6E). Of the remaining *KC* mice, 31% had PanIN2 and 15% had PanIN3 as the most progressed neoplastic pancreatic lesion (Figure 6E). In contrast, none of the *KCI* mice had developed adenocarcinoma by 18 months of age, and PanINs in *KCI* mice were significantly less advanced. Specifically, *KCI* mice exhibited predominantly low grade dysplasia with 67% PanIN1 and 33% PanIN2 lesions at 18 months old (Figure 6E). Interestingly, the lack of progression of low grade dysplasia (PanIN1-2) in *KCI* mice does not significantly prolong the survival of *KCI* mice compared to *KC* mice (Figure 6A). While *KCI* mice develop only low grade dysplasia, by 18 months of age, they have lost essentially all normal acinar cells, and therefore, their death is likely due to pancreatic insufficiency. *KC* mice die at a similar age, due to pancreatic insufficiency and tumor burden. However, the difference in disease progression between *KC* and *KCI* mice strongly supports a promotive role for PKCι in oncogenic *Kras^G12D^*-driven progression of preneoplastic PanIN lesions to malignant adenocarcinoma.

The relationship between the immune system and pancreatic cancer development is complex. Pancreatic inflammation, including immune cell infiltration associated with pancreatitis, can promote the initiation of pancreatic cancer lesion formation [39,49], while subsequent growth and progression of *Kras^G12D^*-driven pancreatic cancer are supported by the recruitment of immune cells that generate a permissive, immunosuppressive tumor microenvironment [50,51,52]. Therefore, we assessed the effect of loss of *Prkci* expression on infiltrating immune cells in *Kras^G12D^*-driven dysplastic pancreatic lesions. The relative levels of immune cells in early pancreatic lesions in 1.5 month old *KC* and *KCI* mice were similar to the differences observed in the pancreata of a 1.5 month old *Prkci^f/f^* and *Prkci*^∆*panc*^ mouse, with significantly more macrophage and T cells associated with *KCI* lesions compared to *KC* lesions (Figure S8A–C). At 18 months of age, immune cell distribution had changed such that similar levels of T cells (Figure 6F) and neutrophils (Figure 6G) were observed in the pancreas of *KC* and *KCI* mice. Significantly fewer macrophages, the major form of immune cell in developing murine *Kras^G12D^*-driven PDAC [53], were detected in the pancreas of 18 month old *KCI* mice compared to *KC* mice (Figure 6H). While there was no difference in the abundance of cells expressing iNOS, a marker of M1 classically activated (pro-inflammatory) macrophages [54], significantly fewer cells expressing ARG1, a marker of M2 alternatively activated (anti-inflammatory) macrophages [54] were observed in *KCI* pancreas (Figure 6I,J), suggesting that pancreatic *Prkci* expression plays a role in the establishment of the immunosuppressive tumor microenvironment permissive for pancreatic tumor progression [50,55,56,57].

### 3.5. PRKCI Expression and Signaling Activity Predicted Poor Survival and were Positively Associated with Autophagy Risk Signature in Human PDAC Tumors

**Figure 7:**
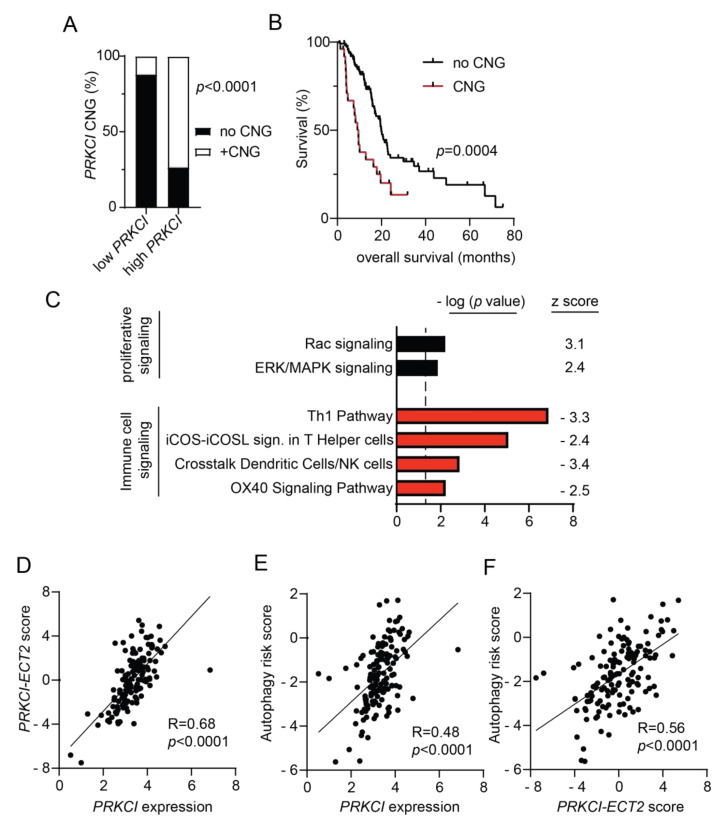
Characterization of *PRKCI* in human PDAC. (**A**) Distribution of tumors without *PRKCI* CNG (GISTIC score −1, 0) and with *PRKCI* CNG (GISTIC score +1, +2) in low *PRKCI* and high *PRKCI* groups defined in Figure S9A. Fisher exact analysis was used to assess the significance of differences between groups. (**B**) Kaplan-Meier analysis of overall survival of PDAC patients surviving >30 days after surgery with *PRKCI* CNG (*n* = 25) and no *PRKCI* CNG (*n* = 113) is plotted. (**C**) Gene expression analysis of the TCGA PDAC dataset identified genes with a significant difference in expression between the 25% PDAC tumors with the highest *PRKCI* expression (*n* = 36) and the 25% PDAC tumors with the lowest *PRKCI* expression (*n* = 36). Ingenuity pathway analysis of differentially expressed genes revealed signaling pathways that are significantly altered between the groups. Bars represent the (−log) *p*-values of the significance of the pathway and the number to the right indicates the z score. Black bars indicated pathways with a positive z score, red bars indicate pathways with negative z scores. (**D**) Scatter plot showing the relationship between log *PRKCI* mRNA expression and the *PRKCI-ECT2* pathway score in all TCGA PDAC. (**E**) Scatter plot showing the relationship between log *PRKCI* mRNA expression and autophagy risk score in all TCGA PDAC tumors. (**F**) Scatter plot showing the relationship between *PRKCI-ECT2* pathway score and autophagy risk score in all TCGA PDAC. Spearman’s correlation coefficient (*R*) and significance (*p*-value) are indicated in panels (**D**–**F**).

Both *PRKCI* mRNA and protein expression levels have been shown to predict poor PDAC patient survival [19,20]. To investigate the clinical relevance of *PRKCI* signaling in human pancreatic cancer, we assessed the association of *PRKCI* expression, *PRKCI* copy number, and signaling activity with PDAC tumor characteristics and patient outcomes. Primary PDAC tumors within the TCGA pancreatic adenocarcinoma dataset were segregated by *PRKCI* mRNA expression level into two groups optimized for survival outcomes. The high *PRKCI* mRNA/poor PDAC patient survival group had a median survival time of 8.7 months while the low *PRKCI* mRNA/better PDAC patient survival group had a median survival time of 19.8 months (*p* < 0.0001) (Figure S8A). We have shown that frequent *PRKCI* CNGs at chromosomal region 3q26 occur in many types of human cancers including PDAC, and CNGs serve as a driver of *PRKCI* expression in PDAC [58,59]. As predicted by our previous studies, PDAC tumors harboring *PRKCI* CNGs were significantly enriched in the high *PRKCI* group (Figure 7A) and PDAC patients with *PRKCI* CNGs experienced significantly worse overall survival than PDAC patients without *PRKCI* CNGs (median survival time 9.6 months vs 19.9 months, respectively; Figure 7B), indicating that *PRKCI* CNG-driven *PRKCI* expression is clinically significant and that *PRKCI* expression and *PRKCI* CNG may be useful as biomarkers of poor patient outcome. 

We recently reported that 3q26 CNGs in PDAC drive coordinate overexpression of *PRKCI* and *ECT2*, a *PRKCI* signaling partner also located on 3q26 [59]. Functionally, PKCι-ECT2 signaling drives two signaling pathways required for the transformed growth of lung cancer cells, RAC-MEK-ERK proliferative signaling, and rDNA transcription and expression of ribosomal RNA processing genes [60,61,62,63]. PKCι-RAC-MEK-ERK signaling also plays a critical role in the transformed growth of PDAC cells [20] and in this report, we show that loss of *Prkci* expression is associated with lower levels of p-ERK in mouse PanINs (Figure S6C), consistent with the association of PKCι and p-ERK expression in PDAC patient tissues [20]. Therefore, we investigated the clinical relevance of the association of *PRKCI* expression with RAC-MEK-ERK signaling in PDAC. Differential gene expression analysis of the 25% of TCGA PDAC tumors with the highest *PRKCI* expression compared to the 25% of PDAC with the lowest level of *PRKCI* expression was performed as described in Methods. IPA analysis of differentially expressed genes revealed that high *PRKCI* expression was positively and significantly associated with active RAC and ERK/MAPK signaling pathways in human PDAC (Figure 7C), strongly supporting the clinical relevance of PKCι-RAC-MEK-ERK signaling in PDAC.

Next, we assessed the clinical relevance of the PKCι-ECT2 driven rDNA transcription pathway in PDAC. We previously reported that a *PRKCI-ECT2* signaling activity score, calculated from a lung squamous cell carcinoma PKCι-ECT2-signaling-dependent gene signature, predicted poor survival of patients with numerous cancer types, including PDAC [59]. As in lung squamous cell carcinoma, expression of genes critical to ribosome biogenesis strongly correlate with both *PRKCI* and *ECT2* mRNA expression in the PDAC dataset (100/286 ribosome biogenesis genes *p* < 0.01 for both *PRKCI* and *ECT2*; see Table S2), suggesting PKCι-ECT2-signaling drives ribosome biogenesis in PDAC. Therefore, we generated a PDAC-specific *PRKCI-ECT2* ribosome biogenesis signature consisting of the top 10% of ribosome processing genes whose expression positively and significantly correlated with both *PRKCI* and *ECT2* expression in PDAC tumors (Figure S9B). We used this gene signature to calculate a *PRKCI-ECT2* pathway activity score for all PDAC tumors in this dataset. The *PRKCI-ECT2* pathway activity score was significantly higher in the high *PRKCI*/poor survival group compared to the low *PRKCI*/better survival group (Figure S9C), and strongly correlated with *PRKCI* expression in the entire PDAC dataset (Figure 7D). Likewise, the *PRKCI-ECT2* pathway score was significantly higher in PDAC with *PRKCI* CNGs (Figure S9D).

We next investigated the association of *PRKCI* expression and signaling with autophagy in PDAC patient tumors. IPA analysis revealed no enrichment of the canonical autophagy signaling pathway in high *PRKCI* tumors compared to low *PRKCI* tumors (-log *p*-value = 0, z score not calculated). However, a PDAC-specific, autophagy-related gene expression signature has been described which predicts poor PDAC patient survival [35]. This PDAC-specific autophagy risk score was significantly higher in high *PRKCI*/poor survival PDAC tumors (Figure S8E) and strongly correlated with *PRKCI* expression over the entire PDAC dataset (Figure 7E). Likewise, the autophagy risk score was significantly higher in PDAC with *PRKCI* CNGs (Figure S8F). Finally, the autophagy risk score was also strongly correlated with the *PRKCI-ECT2* activity score (Figure 7F). Taken together with our preclinical data, we demonstrate for the first time a strong, clinically relevant association between PKCι signaling and autophagy-associated poor survival in PDAC.

High autophagy risk scores were associated with increased macrophage infiltration, but decreased levels of protective immune cells [35], in keeping with the recently described role for autophagy in promoting an immune-suppressive PDAC tumor microenvironment [57,64]. Interestingly, IPA analysis revealed that compared to low *PRKCI* tumors, high *PRKCI* tumors exhibit a significant reduction in several immune cell signaling pathways (Figure 7C). These include the OX40 and iCOS-iCOSL costimulatory signaling pathways which promote T cell survival and activation of T helper cells [65,66]. The Th 1 and “crosstalk between dendritic cells and natural killer (NK) cells” anti-tumor immune signaling pathways [67,68] were also significantly lower in High *PRKCI* tumors (Figure 7C). This strong negative relationship between anti-tumor signaling pathways and *PRKCI* expression suggests high *PRKCI* in PDAC promotes immune suppression. 

## 4. Discussion

*PRKCI* was first characterized as an oncogene in lung and ovarian cancers; its role in other cancer types continues to be described [13,16,18,20,24,62,69,70,71]. We and others have shown that PKCι expression is significantly elevated in oncogenic *Kras^G12D^*-transformed, metaplastic pancreatic acinar cells, suggesting that PKCι plays a significant role in the initiation of pancreatic cancer [22,47]. In this study, we investigated the role of PKCι in pancreas homeostasis, susceptibility to pancreatitis, and initiation and progression of *Kras^G12D^*-driven pancreatic cancer. We report that ablation of PKCι expression in the mouse pancreas altered the pancreatic immune cell population, reduced the severity of caerulein-induced pancreatitis, and blocked *Kras^G12D^*-driven progression of PanINs to pancreatic cancer, revealing for the first time a role for PKCι in the promotion of PDAC in mice.

Knockout of PKCι expression in the mouse pancreas promoted apoptosis, immune cell infiltration, and increased markers of cellular stress. Although *Prkci*^∆*panc*^ mice exhibited a higher level of pancreatic immune cell infiltration, these mice were less susceptible to caerulein-induced pancreatitis. Pancreatitis is a progressive systemic disease with no effective targeted therapy, and severe acute pancreatitis is a risk factor for PDAC [49,72]. The relationship between pancreatitis and autophagy is complex; acute pancreatitis is shown to activate autophagy but also inhibit autophagic flux (reviewed in [73]). Enhancing autophagy activity improved L-arginine-induced pancreatitis [74], while inhibition of autophagy in caerulein-stimulated mouse acinar cells blocked trypsinogen activation [75]. Knockout of the essential autophagy gene, *Atg5*, in the mouse pancreas blocked acinar cell autophagy and reduced caerulein-induced pancreatitis, implicating autophagy in pancreatitis-induced tissue damage [44]. Likewise, our results show that loss of PKCι expression blocked acinar cell autophagy and reduced the severity of caerulein-induced pancreatitis. However, while loss of pancreatic *Prkci* expression induces some of the basal phenotypes associated with the genetic blockade of cellular autophagy, including increased apoptosis, cellular stress, and immune cell infiltration, pancreatic *Prkci* ablation did not reduce overall mouse survival or promote the widespread pancreatic degeneration observed in *Atg5* knockout mice, suggesting PKCι plays a role distinct from *Atg5* in the regulation of autophagy and cellular homeostasis [9,43,76].

Using a mouse acinar cell explant model [77,78], we have shown that genetic or pharmacological inhibition of PKCι significantly suppressed *Kras^G12D^*-induced ADM in ex vivo culture [22]. This suggested that PKCι is required for *Kras^G12D^*-driven initiation of pancreatic cancer, similar to the established role for PKCι in *Kras^G12D^*-initiated mouse lung cancer [16,79]. The unexpected acceleration of *Kras^G12D^*-driven ADM and PanIN formation upon pancreatic *Prkci* ablation in vivo supports a non-cell autonomous role for PKCι in promoting pancreatic tumor initiation. In vivo, pancreatic ablation of *Prkci* blocks autophagy, and like genetic inhibition of autophagy (*Atg5* knockout), *Prkci* ablation promotes increased DNA damage and recruitment of immune cells [9,76]. Interestingly, Zhu et al. recently identified autophagy as an essential, non-cell autonomous metabolic process required for *Kras^G12D^/Trp53^R172H^* mutant mouse pancreatic cancer cell tumor growth [57]. Autophagy was uniquely required for in vivo, but not in vitro pancreatic cancer cell growth, and this requirement was specific for pancreatic, but not lung, tumor growth. Zhu et al. further showed that the dependency on autophagy for tumor cell growth only existed in the presence of an intact immune system, suggesting that a major role of autophagy in pancreatic cancer cells is to promote immune evasion, consistent with reports that an autophagy blockade activates the innate immune response in pancreatic tumors [57,64]. In line with these reports, we show that ablation of pancreatic *Prkci* expression in the *KC Kras^G12D^*-driven PDAC GEMM altered immune cell infiltration of pancreatic lesions. At later time points, *KCI* pancreatic lesions recruited significantly fewer macrophages, a cell type associated with poor PDAC patient survival [80], with a specific reduction in the levels of ARG1^+^ macrophages, an immunosuppressive subset of tumor-associated macrophages [81]. Therefore, the lack of progression of low grade dysplasia in *KCI* mice is likely due to disruption of autophagy and immune suppression that are required for progression of *Kras^G12D^*-driven pancreatic lesions to PDAC [50,82,83]. We further demonstrated that high *PRKCI* expression in PDAC is associated with reduced activation of several immune signaling pathways implicated in the improved survival of PDAC cancer patients, including OX40, iCOS, and Th1 signaling [84,85,86,87].

IPA analysis revealed that high *PRKCI* expression in PDAC was not associated with increased canonical autophagy signaling, suggesting *PRKCI* does not regulate autophagy at the level of gene expression. An extensive proteomic analysis of the human autophagy system characterized an autophagy interaction network under basal autophagy conditions [88]. PKCι was identified as a protein that interacts with multiple ATG8 family members, which are known to play a critical role in autophagosome assembly, maturation and lysosome maturation, and cargo recruitment [88]. PKCι also binds the autophagy cargo adaptor P62 via PB1-PB1 domain-mediated interactions [89]. Therefore, protein-protein interactions and signaling complex formation may be important for PKCι-mediated regulation of autophagy in the pancreas. Future studies will address the mechanism by which *Prkci* regulates pancreatic cell autophagy.

Autophagy is currently being investigated as a novel target for chemotherapy. Preclinical studies in pancreatic cancer GEMMs have demonstrated a therapeutic effect of the autophagy inhibitor, hydroxychloroquine [76,90]; however, hydroxychloroquine as a mono-therapy did not elicit a therapeutic effect in a clinical trial of metastatic pancreatic cancer patients [91]. Recent studies assessing hydroxychloroquine in combination with chemotherapy reveal positive pathologic tumor response in the context of resectable tumors [92,93,94], suggesting more effective inhibitors of autophagy, or combination therapies incorporating autophagy inhibitors, may be clinically useful. A well-characterized inhibitor of PKCι oncogenic signaling, Auranofin, is clinically available and has been previously shown to inhibit transformed growth and growth of human cancer cells as xenograft tumors, including PDAC cells [18,95,96,97]. Since high *PRKCI* expression is associated not only with elevated ERK signaling and high autophagy risk score, but also with lower activities of anti-tumors immune signaling pathways, targeting PKCι may not only inhibit oncogenic signaling, but also reactivate immune protective signaling, such as the T cell activation and survival promoted by the co-stimulatory iCOS and OX40 signaling pathways, and the activation of tumor-targeting NK cells [68]. Interestingly, a recent preclinical PDAC study revealed that reactivation of the OX40 pathway in combination with an immune checkpoint inhibitor induced tumor rejection and immune memory in a mouse model [86]. Another recent study demonstrated that an agonist of CD40 (another co-stimulatory activator of T cells), in combination with inhibitors of MEK and autophagy, activated anti-tumor immunity and inhibited PDAC growth in a mouse model [84] indicating the potential relevance of therapeutically targeting PKCι. The results of this study suggest that further investigation of PKCι-targeted therapy for the treatment of PDAC, perhaps in combination with autophagy inhibitors such as chloroquine, or immune checkpoint inhibitors, is warranted. 

## 5. Conclusions

We previously showed that inhibition of PKCι expression or activity blocks human PDAC cell growth in vitro and in vivo [20,95]. In this report, we demonstrate a previously uncharacterized role for PKCι in the regulation of pancreatic cell autophagy, such that knockout of pancreatic *Prkci* protected mice from *Kras^G12D^*-mediated pancreatic cancer development. *PRKCI* expression and signaling activity exhibit strong positive correlations with poor overall patient survival and an independent autophagy-driven, signature of poor PDAC patient survival, while *PRKCI* expression negatively correlates with multiple anti-tumor immunity pathways, providing clinical relevance to our observation that PKCι plays a promotive role in murine PDAC development.

## 6. Patents

A provisional patent related to this research has been filed (MSB, APF, NRM; Methods and Materials for treating pancreatic cancer, US Patent Application #20110190390).

## Data Availability

The data presented in this study are available in Supplementary Materials.

## Supplementary Materials

The following supporting information can be downloaded at: https://www.mdpi.com/article/10.3390/cancers14030796/s1. Figure S1: Pancreas-specific *Prkci* ablation does not have sex-specific effects on overall survival. Figure S2: Full blots of data presented in Figure 1B. Figure S3: Pancreas-specific *Prkci* ablation does not have sex-specific effects on body weight. Figure S4: Larger images of IHC detection of PKCι expression in pancreatitis tissues. Figure S5: Pancreas-specific *Prkci* ablation does not induce histological alterations or pancreatitis in aged mice. Figure S6: Pancreas-specific *Prkci* ablation does not have sex-specific effects on survival of mice with *Kras^G12D^*-driven pancreatic lesion formation. Figure S7: Pancreas-specific *Prkci* ablation leads to decreased autophagic vesicles and increased zymogen granules in *Kras^G12D^*-expressing pancreas. Figure S8: Immune cell infiltration of PanIN lesions. Figure S9: Characterizing the relationship between *PRKCI* expression, PRKCI-ECT2 signaling and au-tophagy risk score. Table S1: List of antibodies used. Table S2: 286 genes identified as significantly required for pre-rRNA processing.
