# Supplementary material for: Prkci Regulates Autophagy and Pancreatic Tumorigenesis in Mice
Source: Cancers (Basel). 2022 Feb 4;14(3):796. doi: 10.3390/cancers14030796 (PMC8834021; doi:10.3390/cancers14030796)
Supplement: Supplementary file 1 [file cancers-14-00796-s001.zip › cancers-1523563-supplementary/Figures S1-S9 and Table S1.pdf]

cancers

*Supplementary Materials*

# *Prkci* Regulates Autophagy and Pancreatic Tumorigenesis in Mice

**Kristin S. Inman, Yi Liu, Michele L. Scotti Buzhardt, Michael Leitges, Murli Krishna, Howard C. Crawford, Alan P. Fields and Nicole R. Murray**

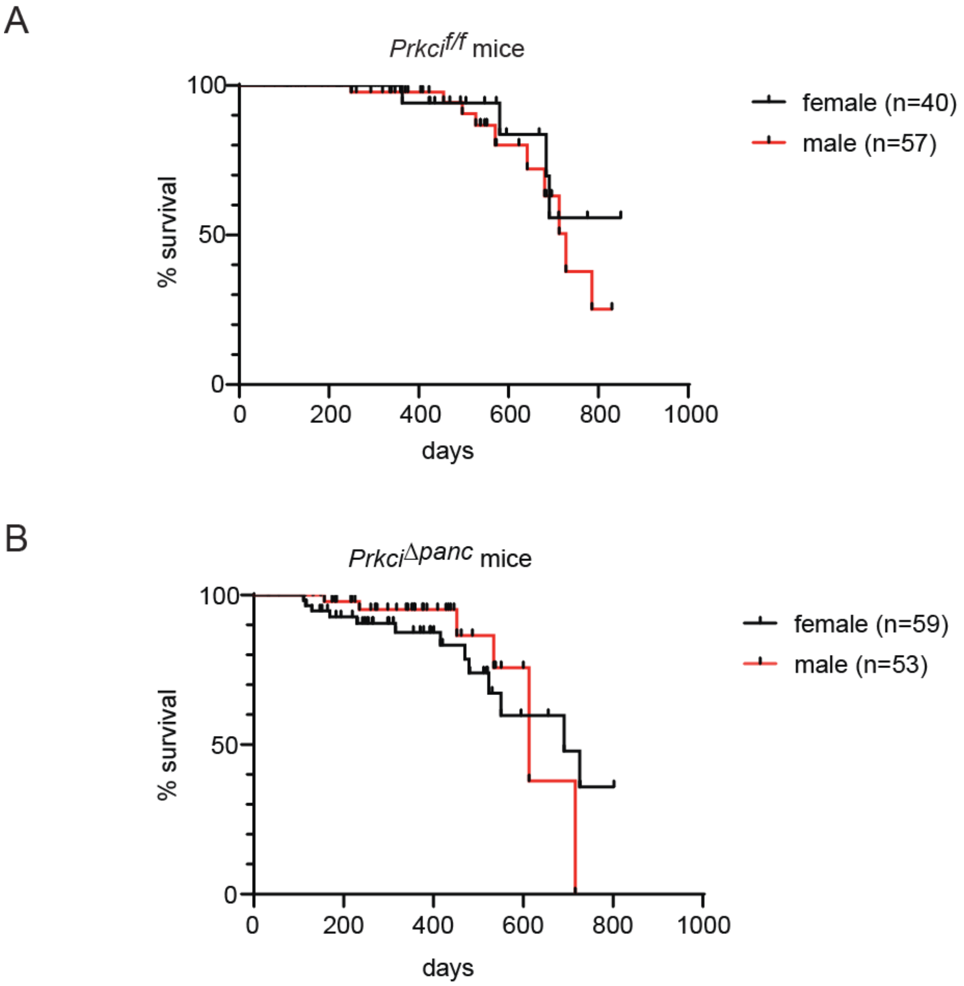


***Figure S1: Pancreas-specific Prkci ablation does not have sex-specific effects on overall survival.*** Kaplan-Meier analysis of overall survival of A) *Prkci*$^{f/f}$ and B) *Prkci*$^{\Delta panc}$ mice plotted by sex. Significance was assessed by log-rank test, there are no significant effect of sex on survival of either genotype.

Full blot Figure 1B; PKCiota blot

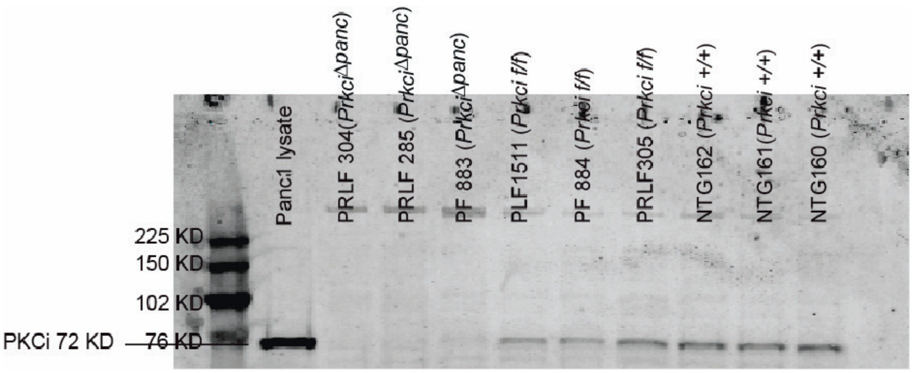


Entire blot is shown, membrane was cut prior to immunoblot analysis to use for another blot

Full blot: Figure 1B; Beta actin blot

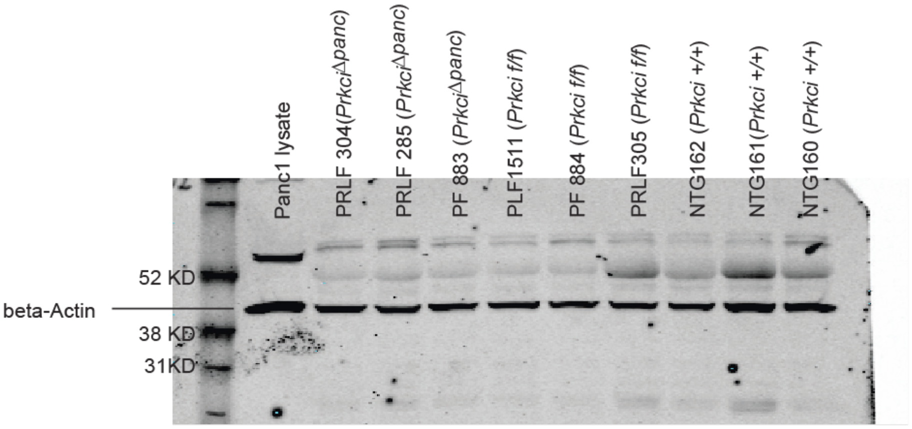


Entire blot shown, membrane cut prior to immunoblot analysis to use for another blot

***Figure S2: Full blots of data presented in Figure 1B.***

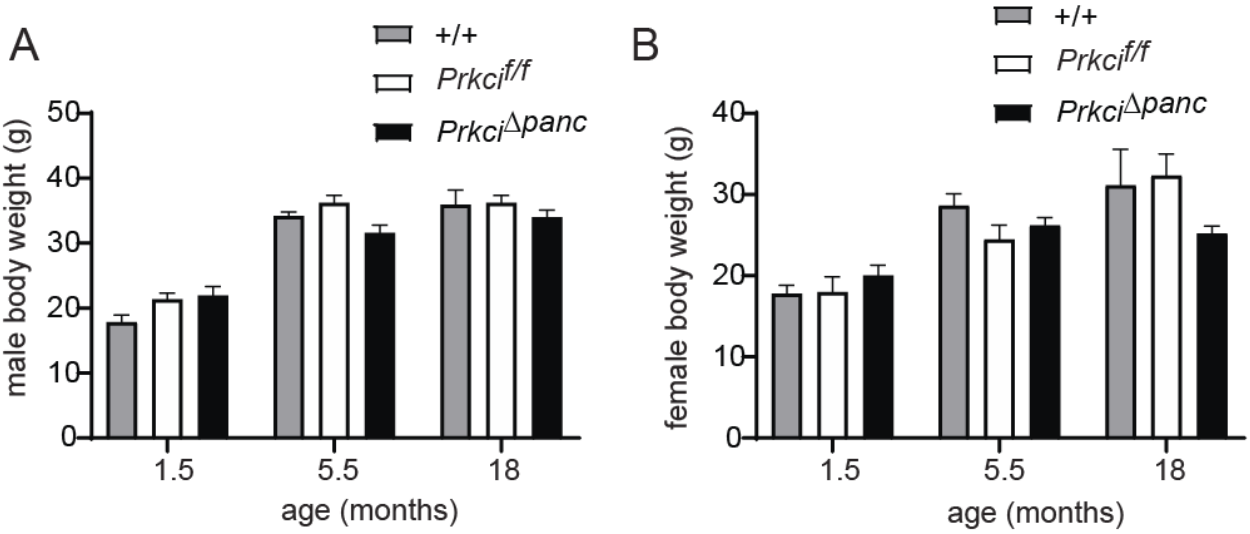


***Figure S3: Pancreas-specific Prkci ablation does not have sex-specific effects on body weight.*** Body weight is plotted for A) male and B) female *Prkci*$^{f/f}$ and *Prkci*$^{\Delta panc}$ mice at three ages. n=≥3/sex/age. Analyzed by 2-way ANOVA. Mouse genotype had no significant effect on body weight for either sex at any of the time points.

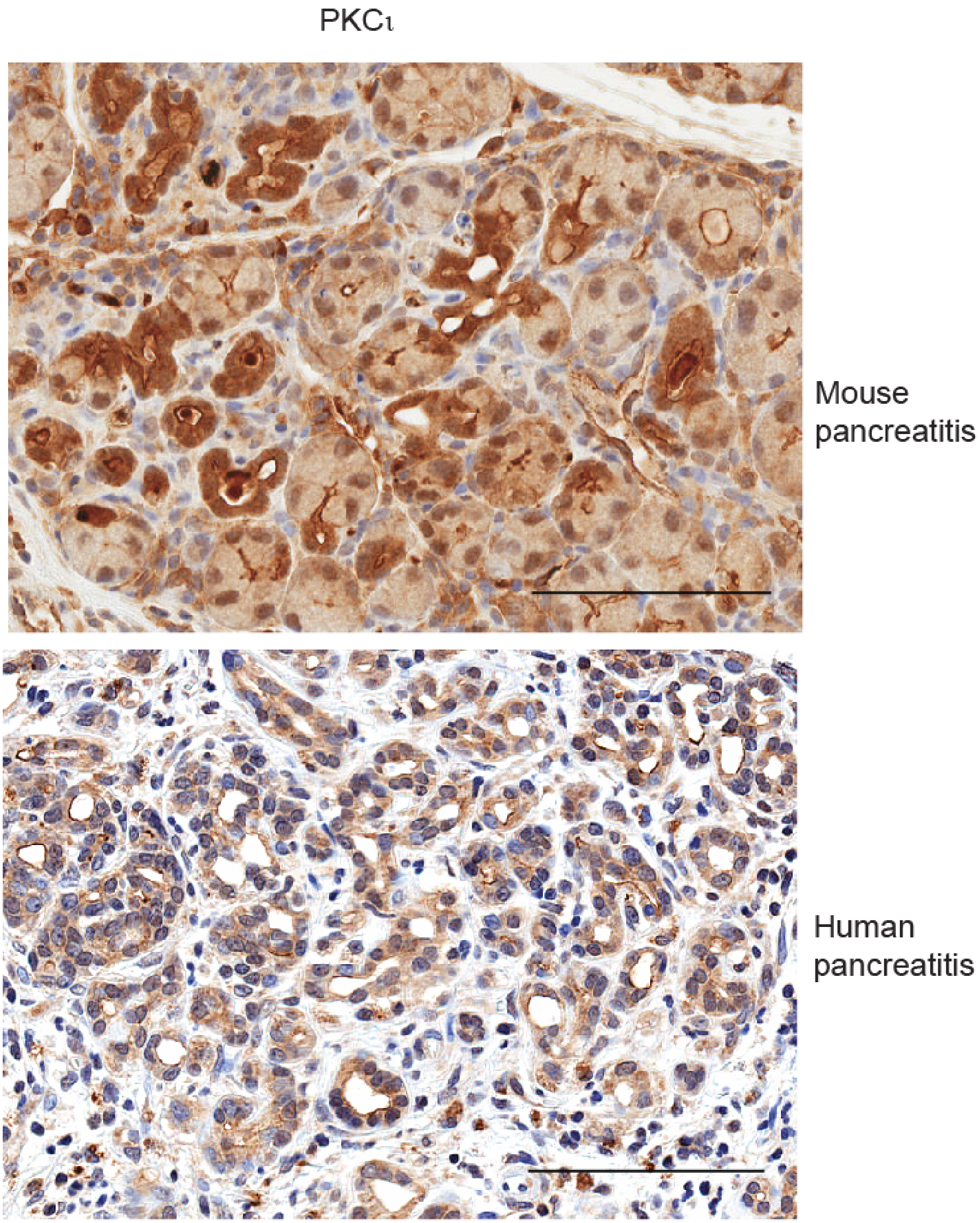


***Figure S4: Larger images of IHC detection of PKCi expression in pancreatitis tissues.*** Top: larger image of mouse severe acute pancreatitis (Figure 3A), bottom: larger image of human pancreatitis tissue (Figure 3A). Scale bars = 100 mm.

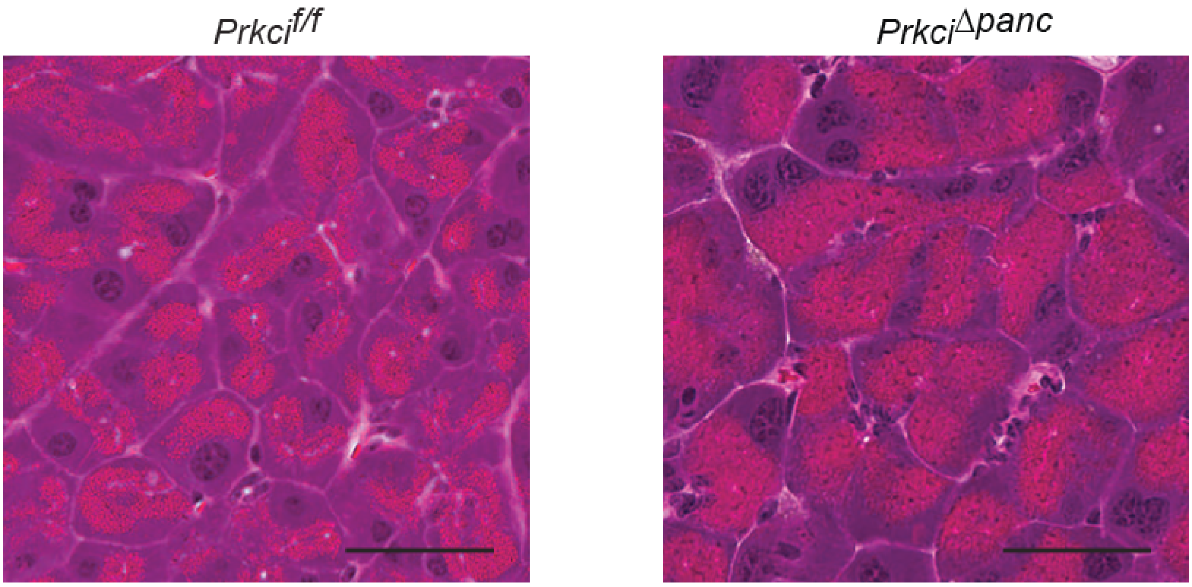


***Figure S5: Pancreas-specific Prkci ablation does not induce histological alterations or pancreatitis in aged mice.*** Representative H&E stained pancreas from 18 month old *Prkci*$^{f/f}$ and *Prkci*$^{\Delta panc}$ mice. Scale bars = 50mm.

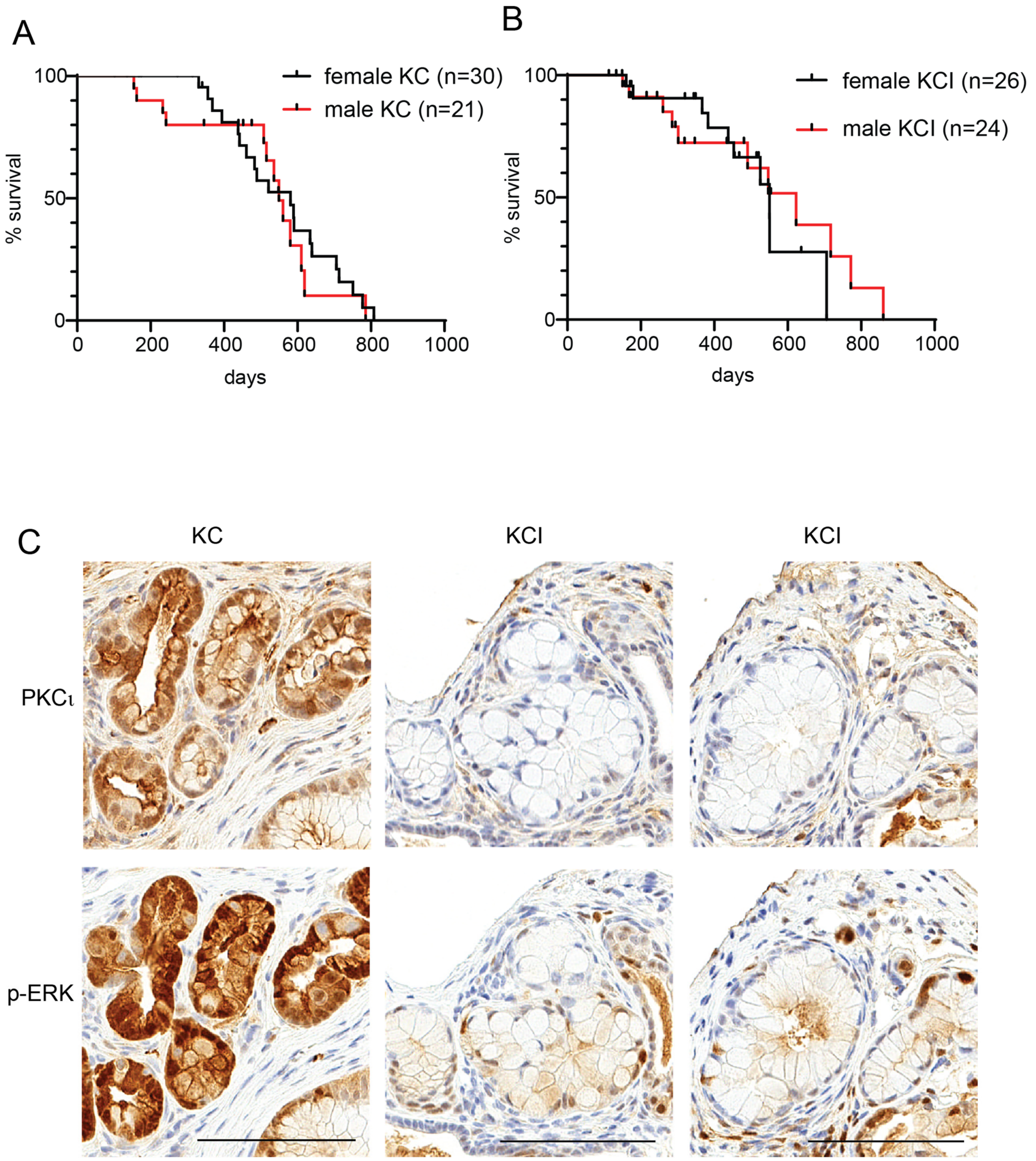


***Figure S6: Pancreas-specific Prkci ablation does not have sex-specific effects on survival of mice with Kras$^{G12D}$-driven pancreatic lesion formation.*** Kaplan-Meier analysis of overall survival of A) *KC* and B) *KCI* mice plotted by sex. Significance was assessed by log-rank test. Mouse sex had no significant effect on survival. C) Representative images of IHC detection of PKCi and p-ERK in *KC* and *KCI* mouse PanIN lesions. Scale bars = 100 mm.

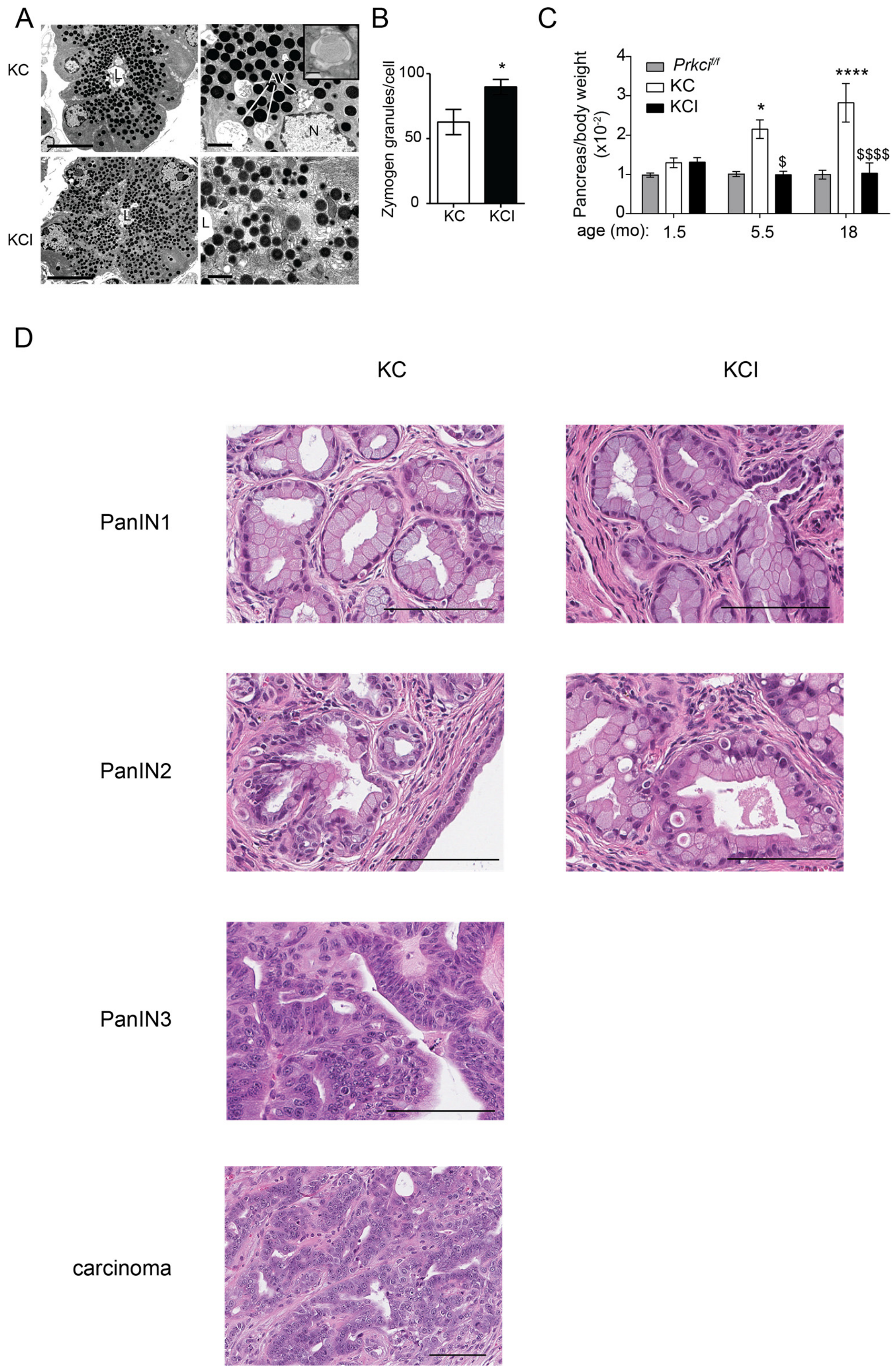


***Figure S7: Pancreas-specific Prkci ablation leads to decreased autophagic vesicles and increased zymogen granules in Kras$^{G12D}$-expressing pancreas.*** A) Transmission electron microscopy of pancreatic epithelial cells of 8 week old *KC* and *KCI* mice at low magnification (left images; scale bar = 10 µm) and high magnification (right images; scale bar = 2 µm). Inset shows double-membrane autophagic vesicle (scale bar = 675 nm). Lumen (L), nucleus (N) and autophagic vesicles (AV) are identified. B) Quantitation of zymogen granules/cell plotted as mean ± SE, n=7 cells/genotype analyzed. *P<0.05. C) Ratio of pancreas/body weight is plotted for *Prkci$^{f/f}$*, *KC* and *KCI* mice ± SE; n≥5, *P<0.05, ****P<0.0001 compared to *Prkci$^{f/f}$*. $P<0.05, $$$$P<0.0001 compared to KC. D) representative images of PanINs and carcinoma in KC and KCI mice, Scale bars = 100 mm.

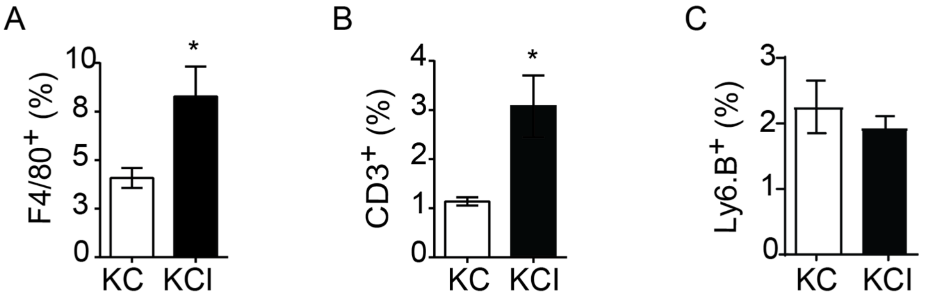


***Figure S8: Immune cell infiltration of PanIN lesions.*** Quantitative analysis of IHC detection of A) macrophages (F4/80), B) T-cells (CD3) and C) neutrophils (Ly6.B) in PanIN lesions of 1.5-month-old *KC* and *KCI* mice plotted as mean ± SE; n=4-5; *P<0.05.

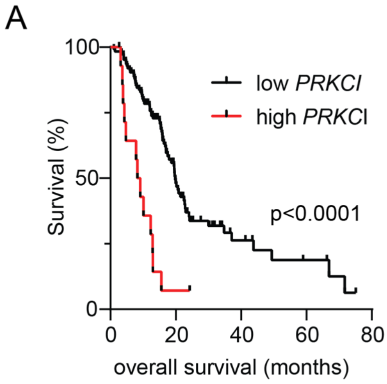


B

| Gene name | Spearman's Correlation (vs *PRKCI*) | p-value | Spearman's Correlation (vs *ECT2*) | p-value |
|---|---|---|---|---|
| RIOK3 | 0.598 | 1.60E-18 | 0.499 | 1.61E-12 |
| SRPK1 | 0.591 | 4.96E-18 | 0.601 | 9.13E-19 |
| XPO1 | 0.579 | 3.27E-17 | 0.493 | 3.02E-12 |
| HNRNPF | 0.531 | 3.08E-14 | 0.484 | 9.26E-12 |
| HEATR1 | 0.497 | 1.97E-12 | 0.391 | 7.26E-08 |
| SPTY2D1 | 0.486 | 7.07E-12 | 0.538 | 1.19E-14 |
| DHX15 | 0.477 | 1.91E-11 | 0.457 | 1.58E-10 |
| PHF6 | 0.469 | 4.66E-11 | 0.398 | 4.25E-08 |
| EIF2AK2 | 0.465 | 6.92E-11 | 0.496 | 2.34E-12 |
| TOP2A | 0.464 | 7.61E-11 | 0.753 | 1.25E-33 |

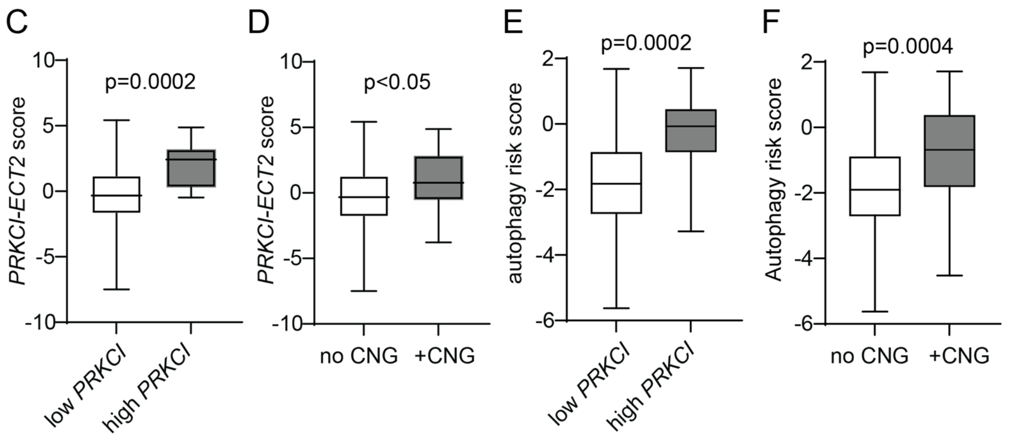


***Figure S9: Characterizing the relationship between PRKCI expression, PRKCI-ECT2 signaling and autophagy risk score.*** A) Kaplan-Meier analysis of overall survival of PDAC patients surviving >30 days after surgery with high *PRKCI* (n=15) and low *PRKCI* expression (n=125) is plotted. B) A *PRKCI-ECT2* activity signature is comprised of the top 10 rRNA

processing genes significantly correlated with *PRKCI* and *ECT2* expression in the PDAC dataset (for full list of rRNA genes and correlations see *Table S2*). *PRKCI-ECT2* activity signature expression was used to calculate a *PRKCI-ECT2* activity score for each PDAC tumor. C) *PRKCI-ECT2* activity score is significantly elevated in PDAC tumors with high *PRKCI* (n=15) compared to PDAC tumors with low *PRKCI* (n=125). D) *PRKCI-ECT2* activity scores are plotted for tumors without *PRKCI* CNGs (no CNGs; GISTIC score -1, 0; n= 118) and for PDAC tumors with *PRKCI* CNGs (GISTIC score +1, +2; n= 26). E) Autophagy risk score [1] is plotted for PDAC tumors with low *PRKCI* (n=125) and PDAC tumors with high *PRKCI* (n=15). F) Autophagy risk scores are plotted for tumors without *PRKCI* CNGs (no CNGs; GISTIC score -1, 0; n= 118) and for PDAC tumors with *PRKCI* CNGs (GISTIC score +1, +2; n= 26). Box plots in panels C-F) represent median, boxes indicate 25% and 75% confidence intervals, and error bars indicate 95% confidence interval. Significance was assessed by unpaired two-tailed t test.

***Table S1: List of antibodies used.***

| Antigen | Company | Catalogue # | Dilution |
|---|---|---|---|
| PKCι | BD Biosciences | 610176 | 1:50 |
| Brdu | Dako | M0744 | 1:8000 |
| Cleaved caspase-3 | Cell Signaling | 9661 | 1:100 |
| CD45 | Millipore | 05-1416 | 1:200 |
| F4/80 | ABD Serotec | MCA497G | 1:150 |
| Ly6B.2 | ABD Serotec | MCA771G | 1:3000 |
| CD3 | Abcam | ab5690 | 1:200 |
| CK19 | TROMA III | DSHB | 1:100 |
| amylase | sigma | A8273 | 1:1000 |
| p-H2A.x | Cell Signaling | 9718 | 1:1000 |
| p62 | Cell Signaling | 5114s | 1:200 |
| FOXP3 | Abcam | Ab54501 | 1:1000 |
| ARG1 | Cell Signaling Tech | 93668 | 1:400 |
| INOS | Cell Signaling Tech | 13120 | 1:200 |
| PD-L1 | Cell Signaling Tech | 13684 | 1:100 |
| insulin | Cell Signaling Tech | 4590 | 1:500 |
| p-ERK1/2 | Cell Signaling | 9101 | 1:100 |
| p-Chk1 | Invitrogen | PA5-34625 | 1:350 |
| Trp53 | Vector Labs | VP-P956 | 1:350 |
| Goat anti-mouse labeled secondary antibody | KPL | 074-1809 | 1:10,000 |
| Goat anti-mouse labelled polymer-HRP | Dako/Agilent | K4001 | |
| Goat anti-rabbit labelled polymer-HRP | Dako/Agilent | K4003 | |

**Supplementary References**

1. Yue, P.; Zhu, C.; Gao, Y.; Li, Y.; Wang, Q.; Zhang, K.; Gao, S.; Shi, Y.; Wu, Y.; Wang, B.; et al. Development of an autophagy-related signature in pancreatic adenocarcinoma. *Biomed Pharmacother* **2020**, *126*, 110080.
